# Supplementary material for: Vitamin D from UV-Irradiated Mushrooms as a Way for Vitamin D Supplementation: A Systematic Review on Classic and Nonclassic Effects in Human and Animal Models
Source: Antioxidants (Basel). 2023 Mar 16;12(3):736. doi: 10.3390/antiox12030736 (PMC10045067; doi:10.3390/antiox12030736)
Supplement: Supplementary file 1 [file antioxidants-12-00736-s001.zip › antioxidants-2229531-supplementary.pdf]

BOLERIAN'S QUERY	RESULTS	EXCLUDED	NOTES
AND ERGOSTEROL ERGOCALCIFEROL			
temperature AND ergocalciferol	61	56	
temperature AND	372	367	
BMD AND ergocalciferol	72	70	
BMD AND ergosterol	2	1	
Immune system AND ergocalciferol	152	150	
Immune system AND ergosterol	196	196	
cognitive function AND ergocalciferol	14	14	
cognitive function AND ergosterol	8	8	
hypertension AND ergocalciferol	116	116	
hypertension AND	6	6	
neoplasms AND	226	226	
neoplasms AND ergosterol	374	368	
Musculoskeletal Pain AND	11	10	
musculoskeletal Pain AND	1	1	
latitude AND ergocalciferol	49	48	
latitude AND ergosterol	5	5	
AND AGARICALES			
Weight gain AND agaricales	49	39	
BMD AND Agaricales	2	2	
cognitive function AND agaricales	23	16	
Hypertension AND	52	48	
immune system AND ergocalciferol AND	2	2	
immune system AND ergosterol AND agaricales	11	10	
Muscoskeletan pain AND ergocalciferol AND	1	1	
Neoplasms AND ergosterol AND agaricales	21	20	
Sunlight AND agaricales	101	80	
UVB AND irradiation AND agaricales	16	9	
UVA AND irradiation AND	8	7	
UVC AND irradiation AND agaricales	5	2	

temperature AND ergocalciferol AND	6	1
temperature AND ergosterol AND agaricales	12	6
AND POLYPORALES		
Weight gain AND	20	20
BMD AND polyporales	0	0
cognitive function AND polyporales	14	14
Hypertension AND polyporales	43	34
immune system AND ergocalciferol AND polyporales	0	0
immune system AND ergosterol AND polyporales	13	13
Muscoskeletan pain AND ergocalciferol AND polyporales	0	0
Muscoskeletan pain AND polyporales	35	30
Neoplasms AND ergosterol AND polyporales	14	11
Sunlight AND Polyporales	25	25
UVA AND irradiation AND Polyporales	1	1
UVB AND irradiation AND Polyporales	4	4
UVC AND irradiation AND Polyporales	0	0
temperature AND ergocalciferol AND Polyporales	0	0
temperature AND ergosterol AND Polyporales	6	6
ALTRO		
Vitamin D2 Enhanced Mushrooms	4	4
Vitamin D Enhanced Mushrooms	25	11
Vitamin D Enriched Mushrooms	22	10
Ultraviolet AND Irradiated AND Mushrooms	78	52

Auriculariales	22	22.00	
Tremellales	108	108.00	
Total n. of papers	2408		
Total n. of papers (without duplicates)	2116		
Total n. of selected papers	98		chosen for review
Total n. of selected papers Phase 2	50		Selected articles about mushrooms enriched by exposure to UV or sunlight
Total n. of selected papers Phase 3	18		Double-blind studies only, on humans or animal models
Papers on human samples	6		
Papers on animal samples	12		
